# Are climate change adaptation strategies working? A call to expedite learning

**DOI:** 10.1111/csp2.70060

**Published:** 2025-05-04

**Authors:** Lara J. Hansen, Deborah A. Rudnick, Kathryn N. Braddock, Arden Drake, Scott Covington, Helen E. Fox, Kimberly R. Hall, James B. Hansen, Carolyn J. Lundquist, Eric E. Mielbrecht, Jordan M. West

**Affiliations:** 1 EcoAdapt, Bainbridge Island, Washington, DC, USA; 2 United States Fish & Wildlife Service, Vancouver, Washington, USA; 3 Coral Reef Alliance, Oakland, California, USA; 4 The Nature Conservancy, Lansing, Michigan, USA; 5 California Department of Fish & Wildlife, Santa Rosa, California, USA; 6 National Institute of Water & Atmospheric Research Ltd, Auckland, New Zealand; 7 United States Environmental Protection Agency, Washington, DC, USA

**Keywords:** adaptation, climate change, climate resilience, conservation, efficacy testing, framework, meta-analysis

## Abstract

Evidence is lacking for what constitutes effective climate change adaptation to successfully conserve and steward ecosystems. Yet we urgently need this information to develop robust adaptation strategies to keep pace with unprecedented change, given our limited resources to do so. This includes not just understanding if a given strategy is effective in a single application, but perhaps more importantly if a given strategy has proven effective across sites where it has been applied, or has benefits only under certain sets of conditions. This learning across the field of adaptation is currently missing and is what is necessary for bringing adaptation to scale. We propose an approach that can guide adaptation efficacy testing under varying levels of baseline knowledge and ecosystem complexity. The approach includes clearly defining conservation goals and climate vulnerabilities, methodically collecting site and climate metrics to inform analysis of efficacy, and evaluating and communicating both positive and negative results in order to advance the adaptation field. Using this approach with meta-analyses and post-hoc testing can quickly scale efficacy testing in a meaningful way. Furthermore, explicitly incorporating efficacy testing into adaptation processes can support the growth of the adaptation field and spark creative, adaptive management approaches that will increase the likelihood of reducing climate change vulnerability.

## 1 | INTRODUCTION

Conservation and restoration efforts increasingly require inclusion of adaptation strategies to reduce climate change impacts and vulnerabilities. The scientific literature on this topic is growing and offers examples of potential adaptation strategies that align with a wide range of conservation management goals (e.g., CCSP, 2008; Heller & Zavaleta, 2009; McLaughlin et al., 2022; Tittensor et al., 2019; Wilson et al., 2020). Several resources have also emerged in recent years that compile case studies and examples of adaptation to support decision making (e.g., EcoAdapt, 2023; Ontl et al., 2020; Tribal Adaptation Menu Team, 2019). The rapid expansion of application and guidance in this area is exciting; however, a recent literature review demonstrates a serious lack of evidence for adaptation efficacy, which is essential for robust decision making (Hansen et al., 2023).

As researchers and managers engaged in climate change adaptation, we formed a working group in 2023 that came together in recognition of a critical need for more (and more comparable) efficacy testing. Our group wanted to create guidance for testing the efficacy of climate change adaptation strategies in order to accelerate learning, which provided the foundation for this manuscript. We define adaptation efficacy as an adaptation strategy’s ability to decrease vulnerability to climate change and deliver desired outcomes or intended effects as evaluated across the field in an experimental context (e.g., comparing no-action controls with implemented strategies); we define adaptation effectiveness as how well an adaptation strategy performs in the field at a given location (after Gartlehner et al., 2006). Given that adaptation is in the early stages of developing as a field of practice, it is not surprising that the science of identifying effective adaptation strategies is similarly nascent and faced with sources of uncertainty (Hoffman et al., 2014; West et al., 2012). Although monitoring and evaluation are often incorporated into conservation practice, these elements are frequently limited to self-reporting or assessing progress toward implementation rather than identifying the effectiveness of specific actions taken (Oakes et al., 2022). In addition, monitoring and evaluation can be early victims of budget constraints or may be curtailed when project funding ends (Biber, 2011; Borja & Elliott, 2013). To meet the challenge of protecting and restoring biodiversity and ecosystem services in the context of climate change, we urgently need to know which solutions work, are cost-effective, maximize use of limited resources, and keep pace with unprecedented change (Nadeau et al., 2024; Pörtner et al., 2022). Conversely, we also need to know what has been tried but failed to achieve the anticipated results (Adams et al., 2014; Catalano et al., 2019).

In order to determine which adaptation strategies represent the best options for reducing climate change vulnerability in ecosystems, we must develop metrics and monitoring approaches that allow us to demonstrate whether these strategies produce desired outcomes. Fortunately, climate change adaptation is not the first topic for which efficacy assessment has been needed, and much can be learned from work testing conservation practice (Bottrill & Pressey, 2012; Geldmann et al., 2015). For example, adaptive management guides conservation management decisions by monitoring decision points for evidence of success (or lack thereof) (Wilhere, 2002); and experimental design techniques such as before-after-control-impact (Benedetti-Cecchi, 2001) assess whether protected areas effectively support biodiversity (Smallhorn-West et al., 2020; Wauchope et al., 2022). However, some broadly replicated strategies, such as coastal restoration projects, may have few instances of monitoring to assess their success, risking repeated inefficient allocation of resources on strategies that are potentially ineffective (Bayraktarov et al., 2016). To date, the field of climate change adaptation as a whole has also shown a lack of efficacy assessment (Giffin et al., 2020; Hansen et al., 2023; Wilson et al., 2020). As a result, the field needs an approach for measuring efficacy of climate change adaptation strategies. Lacking such an approach risks failing to identify adaptation strategies that may result in maladaptive outcomes, diminishing effectiveness over the long term, or causing concomitant disadvantages under the false assumption that strategies are beneficial.

As we advance our understanding of which adaptation strategies are effective, there is value in identifying how and where we can most readily assess efficacy. Opportunities should be found to build our knowledge base more rapidly by conducting meta-analyses on groups of comparable actions within a strategy (e.g., use of living shoreline projects to reduce the impacts of sea level rise (Huynh et al. 2024)). In the following sections, we outline an approach to assessing adaptation efficacy and discuss key elements for its application. It should be noted that many existing adaptation frameworks, such as Climate Smart (Stein et al., 2014), Steps to Resilience (Gardiner et al., 2022) and resist–accept–direct (Lynch et al., 2022), have monitoring or evaluation steps, and other frameworks have been proposed for identifying indicators of effectiveness (Pearce-Higgins et al., 2022; Peterson St-Laurent et al., 2022). These frameworks primarily describe the process of designing and implementing adaptation projects. When monitoring efforts are described, they typically focus on evaluating the success of an individual action within the context of a specific location, not for broader suitability of a strategy. While such data are the backbone of effective adaptive management and an essential element for meta-analysis in the approach described in this paper, monitoring and evaluation in these existing frameworks is not focused on developing a field-wide evidence base for adaptation practice by evaluating efficacy across sites to establish suitability for application more broadly. Hence, the creation of this complementary approach.

## 2 | IDENTIFYING TESTABLE ADAPTATION STRATEGIES

In order to assess the effectiveness or efficacy of adaptation actions or strategies, several key elements should be clearly defined at the outset. These include defining over-arching objectives and conservation targets, identifying climate change risks and vulnerabilities, articulating adaptation strategies that address those vulnerabilities, outlining climate-smart design and implementation methods, and creating a detailed plan for disseminating information gained from the assessment. Additionally, it is critical to use replicable methodology to collect and store data for evaluating the strategies (BOX 1).

**FIGURE 1:**
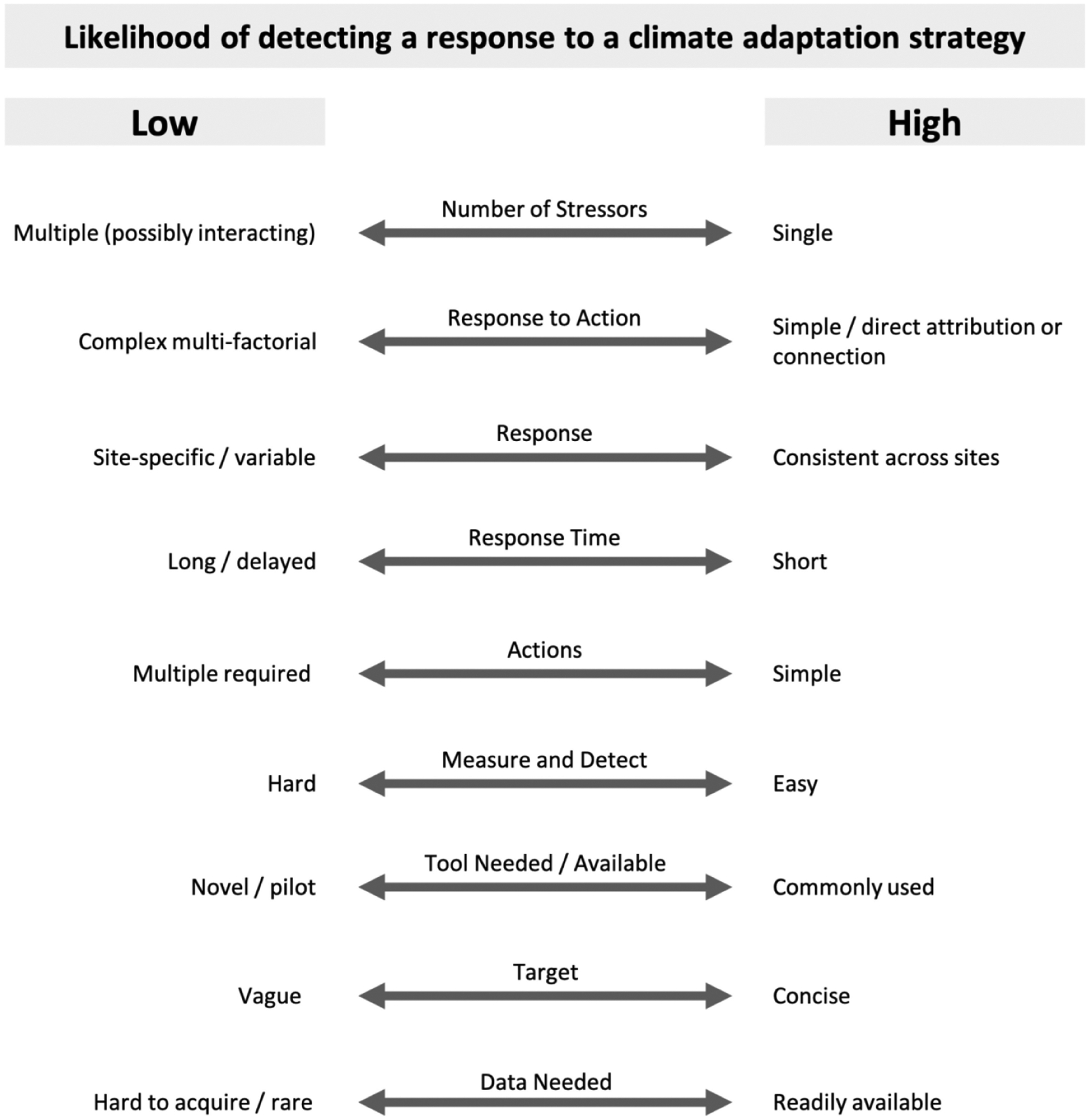
Factors influencing the likelihood of detecting a response to a climate adaptation strategy.

Multiple factors influence the likelihood of detecting a response to a climate change adaptation strategy, including the number of interacting stressors, the complexity of the ecological response, geographic extent, and response time (Figure 1). Detecting a response can also become challenging due to overlapping effects when multiple strategies are needed. By contrast, clear targets, easily obtainable measurements, and the use of familiar tools all increase the likelihood of response detection. Additionally, intensive data collection can enhance the probability of accurately attributing observed responses. For instance, continuous monitoring of ecosystems (e.g., through satellite remote sensing) can provide more robust evidence of efficacy and better document incremental changes in restoration efforts compared to sporadic data collection; however, the intensity of data collection needs to be balanced with a feasible and affordable level of effort.

**FIGURE 2:**
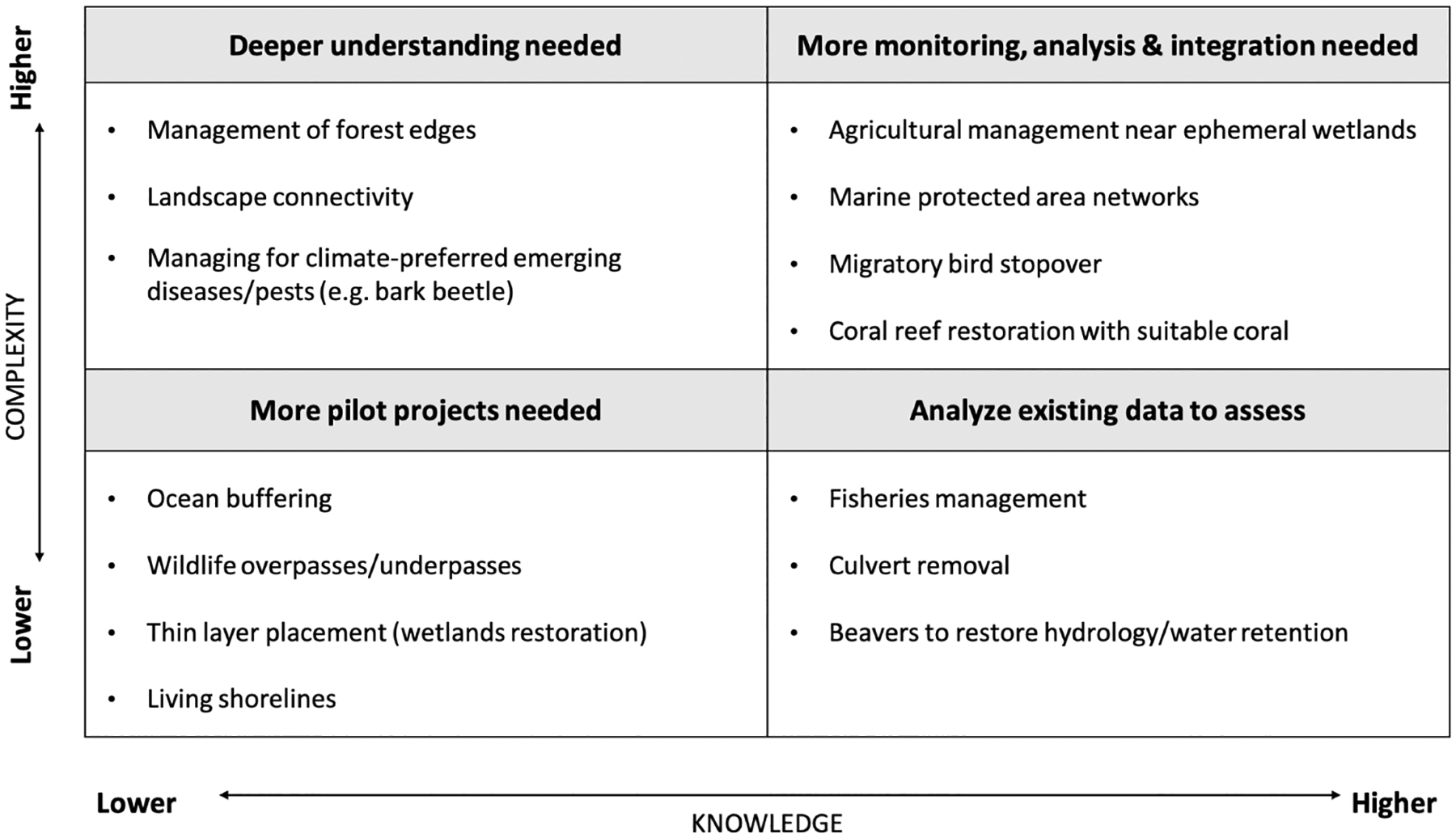
Identifying adaptation strategies to prioritize for testing can be informed by the relative complexity of the issue and existing knowledge (e.g., monitoring data, systems understanding). These examples from conservation biology and their placement in the knowledge and complexity space was determined by a group of experts who work in conservation and climate adaptation, and provide a generalized illustration of how present-day suitability for efficacy testing success varies across strategies and the categories of approaches that would be needed for each combination of complexity and knowledge.

When determining how testable an adaptation strategy might be, system complexity should be considered together with the state of knowledge for a given adaptation topic area (Figure 2). Complexity increases along with the number of interactions and interdependencies in the ecosystems where adaptation strategies are being applied and can also be associated with various challenges that emerge during implementation and monitoring. Evaluation of the state of knowledge could include literature reviews, interviews, determining whether pilot studies have occurred, and whether extant data can be used to gain insights from past responses to similar strategies. Ranking complexity and knowledge in order to group adaptation strategies can help focus on productive areas for efficacy research (Figure 2). In the case of low complexity and high knowledge, there is a wealth of information, and the system is relatively simple to understand, which results in ample data for assessing the efficacy of adaptation strategies. By contrast, where complexity is high and knowledge is low, there is a need for a deeper understanding before proceeding with efficacy assessment. In the other two cases (low complexity/low knowledge and high complexity/high knowledge), efficacy could be explored through the application of more pilot projects to test these strategies or more monitoring and analysis of existing applications across multiple fields, sites, and scales. In all cases, there is almost certainly the need to overcome technological barriers, assess conflicts and trade-offs, and address barriers to resource and/or institutional capacity (Neveu et al., 2018; Stein, 2024).

Adaptation strategies can have unintended consequences, including maladaptation wherein unintended outcomes may undermine desired outcomes (Lukasiewicz et al., 2013; Schipper 2020) or adversely impact a non-target aspect of the system. This risk may be greater when system complexity is high, creating challenges for a priori knowledge of a full range of outcomes (Ingwersen et al., 2013). Considerations of unintended outcomes in climate change adaptation may include recognizing the possibility for shifts in populations and resources that are not the direct target of the strategy (Szuwalski et al., 2023), explicitly incorporating uncertainty and complexity through techniques such as anticipatory framing and scenario planning (Larson et al., 2015; Miller et al., 2022; Suckling et al., 2021), and potentially adjusting implemented actions in light of novel results (Hansen & Hoffman, 2011). Acknowledging such possibilities prior to and during strategy implementation can strengthen efficacy research.

## 3 | AN APPROACH FOR TESTING ADAPTATION EFFICACY

To move the field of adaptation from practices built on assumptions to practices built on evidence requires systematic collection of information and the use of that information to support analysis and learning (Hoffman & Hansen, 2022; Singh et al., 2020). This information should include when, where, why, and how to implement adaptation projects and programs, as well as who defines, implements, and benefits from adaptation strategies. Applying this approach is most directly suited to those attempting to assess the efficacy of adaptation strategies as part of effectiveness research or implementation of an adaptive management approach (see SI1: Worksheet of questions to measure the efficacy and effectiveness of adaptation).

**FIGURE 3:**
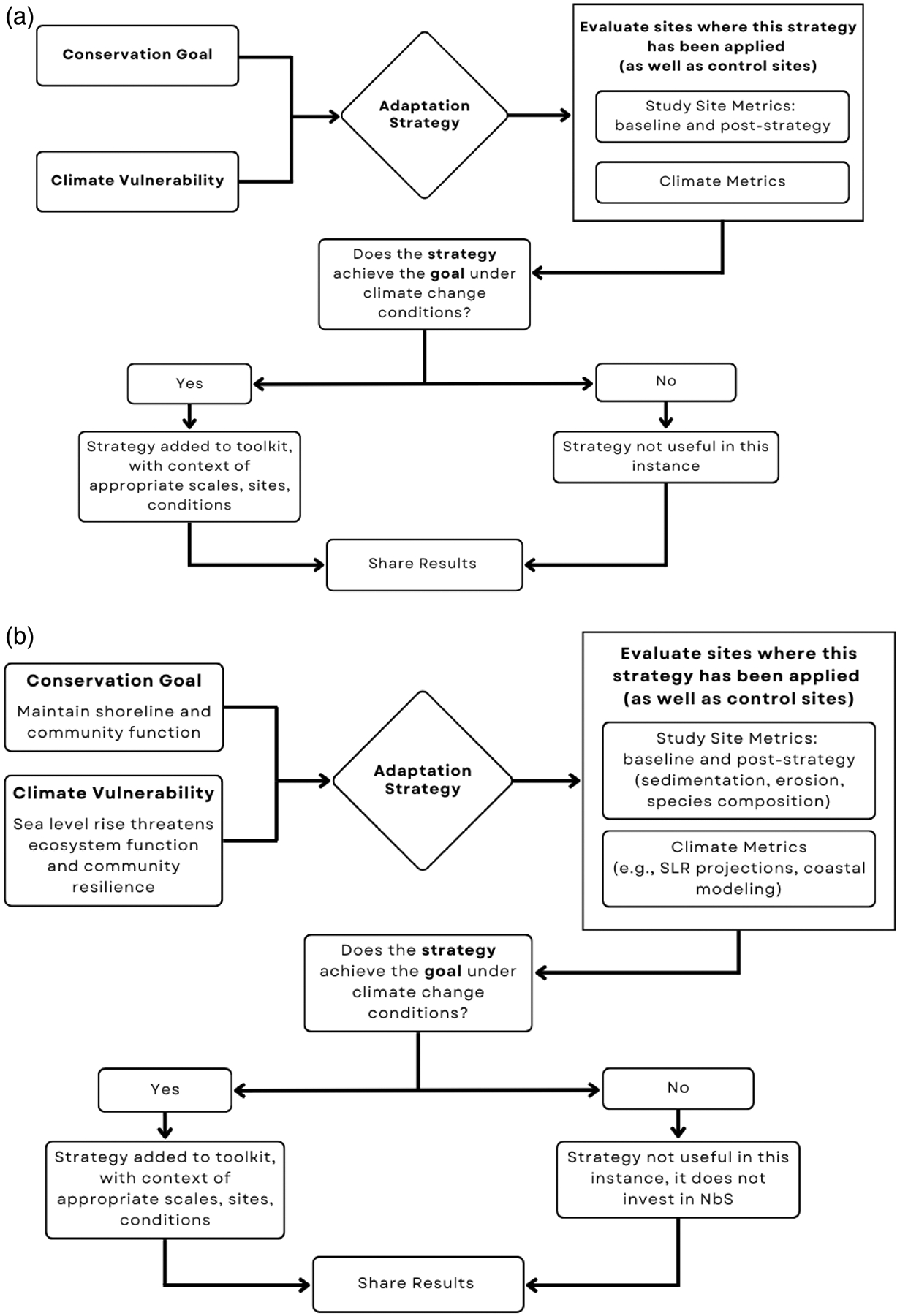
(a) Adaptation efficacy testing flowchart. (b) Adaptation efficacy testing flowchart showing an example for testing a strategy designed to maintain shoreline and community functions.

This approach is conceptualized as a flowchart (Figure 3a, b, in which (b) provides an applied example). The climate vulnerabilities that are likely to affect the conservation goal need to be identified. Adaptation strategies are designed to support or modify the original goal in order to reduce the vulnerability. To evaluate an adaptation strategy (and ideally, compare it to “no action” control or reference sites), the site and climate metrics identified should be ones for which data are available, and which will have a high likelihood of detecting change. Analysis of these data can be used to determine if the strategy decreases system vulnerability under changing climate conditions compared to the control site. If the strategy reduces vulnerability under changing climate conditions, it should be added to our collective toolkit of effective adaptation strategies under the conditions tested. If the strategy does not reduce vulnerability under changing climate conditions, then it is not useful in this instance and should be identified as such. Additional testing may indicate usefulness under different degrees of climate exposure, or in a different ecosystem type or landscape context. However, if a strategy shows no evidence of efficacy after multiple tests under a variety of conditions, it can be set aside and replaced with something else.

Finally, it is essential to communicate findings to fellow practitioners: Cogent, timely, and broad transmission of results is critical to ensure that the adaptation field’s knowledge base continues to grow. This approach is purposefully kept general so that it can be utilized across a variety of geographies and types of adaptation, facilitating learning even when the specific applications differ.

Table SI2 outlines three example applications of the approach. One example considers stream restoration with a conservation goal to improve water quality in freshwater aquatic and riparian habitats to benefit aquatic organisms such as fish (Table SI2). Climate vulnerabilities that may impact the ability of managers to attain this goal likely include warming air temperatures (including extreme heat and heat waves) and lower streamflows (as a result of precipitation changes, and drought interspersed with greater storm intensity). These are factors that increase stream temperatures and sedimentation during high flow/scouring events, impacting fish and other aquatic organisms. To address these vulnerabilities and help achieve the goal of improved water quality, a commonly recommended strategy is to increase riparian canopy cover to shade streams, intercept and reduce scouring overland flow, and reduce water temperatures. To assess the efficacy of this strategy, researchers, including managers, could compare sites where tree planting efforts have restored canopy cover over the stream channel with unrestored sites and potentially with undegraded sites that maintained intact canopy cover in the absence of degradation. Researchers would look at a range of indicators and metrics associated with study sites (e.g., % canopy cover, channel morphology, presence of bioindicators associated with high water quality such as macroinvertebrates) and climate-related factors (e.g., instream temperature, air temperature, precipitation, stream discharge and to model longer-term outcomes, climate projections) to determine whether tree planting within riparian areas increased stream shading, resulting in lower water temperatures and improved water quality. Evaluation of other metrics including species composition and tree survival rates in restored areas could provide additional information about site conditions and planting practices that maximize project success.

The supplemental information presents additional examples of how the approach could be employed to address other specific adaptation challenges, including hydrologic restoration using beavers or beaver dam analogs, and building coastal resilience with nature-based solutions (see SI2: Examples of worksheet question answers to measure the efficacy of adaptation).

## 4 | AMPLIFYING EFFICACY TESTING: THE ROLE OF META-ANALYSIS AND POST-HOC EFFICACY TESTING

While there is significant value in monitoring and evaluating individual sites and the actions taken there, the urgency of adaptation requires that we also maximize the power of results by assessing strategies across multiple sites, such as by meta-analysis across multiple sites that have already implemented common adaptation strategies. It is possible to identify strategies that are more conducive to such analysis by starting with those with low complexity and high knowledge (Figure 2) and examining factors that are more easily detected (Figure 1). This post-hoc approach to evaluation can allow for more rapid learning, including reflection on effectiveness over time and through extreme events.

In some cases, datasets for post-hoc testing and meta-analysis already exist. For example, publicly available data exist for water quality monitoring (Hutton et al., 2022; Riskin & Lee, 2021), species composition, changes in predicted habitat, and remote sensing (e.g., water temperature, primary productivity). Habitat mitigation and restoration projects represent another potential data pool for assessing adaptation efficacy, including wetland creation and salt-marsh rehabilitation for flood control, living shorelines, and riparian habitat restoration, all of which are testable with the approach proposed here. These projects also generally require annual monitoring, which is collected in reports that can be accessed and used for meta-analyses. Meta-analyses of multiple sites addressing a common strategy or set of strategies could be further facilitated by establishing standardized sets of monitoring questions and metrics (Evju et al., 2020).

## 5 | WE MUST LEARN FASTER

Development and application of approaches for adaptation efficacy testing is part of a culture shift necessary for achieving effective climate change adaptation. This shift requires that meaningful adaptation strategies, including novel approaches, are implemented and evaluated both individually and across multiple sites. Harnessing the power of meta-analysis, as we have described, is but one approach. We recognize scaling up is hard and can feel risky. Here, we share some thoughts on how our community of adaptation in practice can increase the pace of progress and learning.

- Taking a thoughtful action, informed by climate change, is likely to be less risky than taking a path of inaction (or status quo) and hoping for the best. In cases where some form of constraint (social and financial) prevents action, the “no action” alternative is useful as a potential reference treatment; fully documenting the “no action” control and its outcomes can be very useful to generate evidence for comparison with adaptation strategies implemented at other locations.
- “No regrets” actions are a good place to start. These are based on the best available information that is highly likely to be effective and has a low likelihood of harm or impeding other needed actions (e.g., reducing pollution in coastal waters, preventing deforestation). Even if these actions are not ultimately demonstrated to effectively reduce climate change vulnerability, they may be warranted based on co-benefits such as public health or water quality improvements. Of course, the cautions discussed above regarding unintended consequences still apply, and a robust efficacy testing approach should account for monitoring unintended outcomes.
- It is of paramount importance to document both successes and failures. By sharing results, especially negative results with collaborators, as well as through peer-reviewed literature, gray literature, practitioner networks, and research collaboratives, we can increase access to an adaptation evidence-base (Arechavala-Gomeza & Aartsma-Rus, 2021; Catalano et al., 2019; Dickson et al., 2023; Hansen et al., 2023).
- Where adaptation projects are not effective, it is possible that the original target, adaptation strategy, and/or the scale of the action taken may be insufficient to address the degree of changes occurring. In this case, the design, scope, and/or target should be revisited, such as with the Resist-Accept-Direct framework (Schuurman et al., 2022) wherein practitioners assess the most appropriate strategy within a continuum of management approaches. This method also considers whether the original target might ultimately be unrealistic or unattainable, and a different management goal might become appropriate. Maladaptation could also be identified by noting unintended, nonadvantageous consequences.
- Scale should be explicitly considered in selecting adaptation strategies and designing their assessment (Stein, 2024). Simply assuming the scale of an action is large enough may be insufficient. For example, the Great Barrier Reef Marine Park was at one time considered to be less vulnerable to coral bleaching based on the protected areas’ immense size and good management (Wilkinson, 2000). However, increasing water temperatures in subsequent years resulted in bleaching levels consistent with smaller protected areas (Day, 2018; Virgen-Urcelay & Donner, 2023).
- Care should be taken to ensure that adaptation strategies are designed to address the climate impacts identified in the vulnerability assessment stage, not just strategies that are convenient to implement or already in the user’s toolkit. The practitioner should document their decision process and design specifications so that it is possible to track what the strategy is meant to be addressing and how that is being accomplished.
- Projects that integrate benefits to people can complement resource-focused monitoring data with interviews and short surveys (Bailey et al., 2020) to capture additional elements of project effectiveness and community support.
- Building a library of efficacy-tested adaptation examples and datasets can provide a foundation for prioritization of adaptation strategies based on demonstrated success. Robust examples already exist in the conservation field and can provide useful templates (Collaboration for Environmental Evidence, 2021; University of Cambridge, 2020).

## 6 | CONCLUSIONS

We propose that this approach to efficacy testing of adaptation strategies be applied broadly, and results shared with conservation and resource management networks, researchers, and existing or emerging monitoring networks (e.g., soil monitoring, water quality monitoring, remote sensing), to rapidly expand the evidence base needed to support the implementation of management and restoration activities that are likely to reduce climate change vulnerability. While the approach presented here was designed specifically for adaptation strategies focused on natural resources management and conservation, it could also be used to assess the efficacy of adaptation practices in any field (e.g., local planning, public health, environmental justice, transportation).

The integration of monitoring and evaluation into project design to facilitate the assessment of efficacy should be done collaboratively to ensure that it is successful and completed at a scale that will allow meaningful conclusions to be drawn. For instance, practitioners who are unable to undertake monitoring, data collection, and analysis may benefit from being connected to researchers who can support these activities. For topics that lend themselves well to evaluation (e.g., less complex systems where there is sufficient knowledge), meta-analyses that use existing monitoring data to compare the outcomes of similar adaptation actions can allow rapid gains in our understanding of adaptation efficacy. Funders and funding programs can also be essential partners in main-streaming monitoring and evaluating adaptation efficacy through the use of approaches such as these, as they are well positioned to encourage or require efficacy testing from their grantees and networks that they support.

## Supplementary Material

Supplement1

SUPPORTING INFORMATION

Additional supporting information can be found online in the Supporting Information section at the end of this article.

## Data Availability

The manuscript involved no original data collection or analysis.

## BOX 1 Required factors for ensuring that the efficacy of adaptation efforts is measurable.

- **Multiple treatment and control sites** to allow adaptation strategies to be compared more experimentally (e.g., by comparing the same strategy applied across multiple locations with control sites at each location where no action is taken)
- **Clear connection between metrics and climate vulnerabilities** to ensure that evaluation of adaptation strategies is consistently framed within the context of the vulnerabilities they are designed to address
- **Documentation of methodology** (e.g., process of site selection and project evaluation) **and adaptation actions implemented** to ensure that the assessment can be replicated
- **Datasets that already exist or can be collected**, ideally housed in a location that will make them accessible for building knowledge across multiple locations and adaptation strategies
- **Communication of results and replicable methodology** through the distribution of this knowledge to other practitioners and researchers with the goal of building an evidence base for the field of adaptation
