# Supplementary material for: Are climate change adaptation strategies working? A call to expedite learning
Source: Conserv Sci Pract. Author manuscript; Available in PMC 2026 Jun 10. (PMC12186239; doi:10.1111/csp2.70060)
Supplement: Supplement1 [file NIHMS2090015-supplement-Supplement1.docx]

**Supplementary Information #1: Worksheet of questions for measuring the efficacy or effectiveness of adaptation**

Overarching goal: Climate adaptation action is urgently needed, but for many conservation adaptation strategies and actions, there is little tested evidence of efficacy (field-wide testing) or effectiveness (site-specific testing). In order to make recommendations, we need improved information on what is actually conferring real benefit. This worksheet can be adapted to evaluate the efficacy or effectiveness of adaptation strategies or actions. In Supplementary Information #2, an example is provided of how the worksheet can be applied to assess the efficacy of strategies. (Note: This worksheet was designed for conservation but can be applied to any topic.)

| I. Conservation target/goal: |
| --- |
| II. Climate vulnerabilities: |
| III. Adaptation taken to reduce those vulnerabilities: |
| IV. Efficacy assessment objective(s): |
| V. Identification of project sites and data sources   - Positive and/or negative control sites (include how these sites were selected) - Monitoring/study sites - Baseline conditions (physical condition and selection criteria) |
| VI. Metrics and indicators   - Project objectives - Study site parameters (e.g., topography, acreage, species composition) - Climate parameters (e.g., temperature, precipitation, pH) |
| VII. Data collection   - Timing and frequency for data collection (consider time horizon for metrics & indicators) - Extant data sets to leverage, if any? |
| VIII. Data analysis. How will the data be used to determine efficacy? |
| IX. Results. Determination of efficacy, and additional considerations as relevant including:   - Magnitude of the effect of the action - Rate of improvement - Geographic applicability - Benefit of the actions (or return on investment) |
| X. Dissemination and communication   - Audience(s) - Programmatic or jurisdictional requirements? - Reporting interval and methods |
| XI. Conclusions: What recommendations can be drawn from the results of the proposed assessment? |

**Supplementary Information #2: Examples of worksheet question answers to measure the efficacy of adaptation**

This table shows three examples of how the proposed worksheet would be used to support improved monitoring and evaluation practices that would allow efficacy testing for these strategies.

| I. Conservation target/goal | **Example #1: Improve mountain meadow ecosystems through increased hydrologic resilience and floodplain connectivity** | **Example #2: Enhance freshwater aquatic and riparian habitat by improving water quality** | **Example #3: Increase coastal ecosystem resilience through nature-based solutions (NbS)** |
| --- | --- | --- | --- |
| II. Climate vulnerabilities | Forest meadow landscapes in the western U.S. are increasingly vulnerable to climate change impacts that impact hydrology, including changes in precipitation amount and timing, drought, and flooding. | Warming air temperatures (including extreme heat or heat waves) and reduced streamflow (from changes in precipitation patterns and drought) can increase stream temperatures, impacting fish and other aquatic organisms. | Coastal ecosystems are increasingly threatened by climate change impacts, including sea level rise and increased storm intensity and frequency. |
| III. Adaptation action(s): How the action(s) reduce climate vulnerabilities | Conservation/reintroduction of beavers or analogs can address impacts through restoration of hydrologic and habitat benefits. | Increased/improved riparian forest canopy can help maintain cooler stream water temperature by providing shade and reducing direct sunlight. | Implementation of nature-based solutions (NbS) in a Resist-Accept-Direct framework to meet ecological and human needs for coastal resilience. |
| IV. Efficacy assessment objective(s) | Measure efficacy of beaver reintroduction or beaver dam analogs to achieve hydrologic objectives (e.g., increased water quality and supply, floodwater storage, hydrologic connectivity) | Measure the efficacy of riparian forest canopy restoration in regulating climate-driven increases in stream temperatures | Measuring the efficacy of NbS in maintaining and enhancing shoreline integrity as climate changes |
| V. Identification of project sites and data sources   - Control sites (positive/negative) - Monitoring/study sites | - Remote sensing to establish comparable hydrology across candidate sites - Multiple current sites & projects for meta-analysis | - Intact riparian forests with ample canopy cover vs. degraded/unrestored riparian areas with little canopy cover - Ensure streams are of a similar order and placement within the watershed - Select sites where monitoring is already in place (e.g., USGS stream gages) | - USNERRs, NPS, and USFWS have multiple sites with and without NbS that can be compared and evaluated - Large site networks are ripe for monitoring, meta-analyses |
| VI. Metrics and indicators for the following (baseline and over time):   - Study site parameters - Climate parameters | - Study site parameters: physical (total wetted area, depth of channel incision); chemical (total suspended solids, sediment load); biological (invertebrate taxa, vegetative composition) - Climate parameters: air and water temperature, precipitation, streamflow, climate models to evaluate persistence | - Study site parameters: percent canopy cover (remote options: GIS, drones); channel morphology; bioindicators associated with water quality (e.g., macroinvertebrates) - Climate parameters: in-stream temperature (continuous; monthly averages), air temperature, precipitation, stream discharge | - Study site parameters: sediment accretion rates; wave attenuation; coastal erosion rates; marsh integrity; interior ponding, hydrologic integrity; changes in species composition (possible early warning indicator) - Climate parameters: ocean temperatures, sea level, storm frequency, ocean acidification |
| VII. Data collection   - Timing & frequency for data collection - Consider time horizon for metrics & indicators - Extant data sets to leverage, if any? | - Seasonal and annual after introduction, through extreme weather events (e.g., flood, drought) - Measure before and after project, control, and impact (BACI) - Organizations with potential data sets: California Dept of Fish & Wildlife, Wildlife Conservation Society, US Forest Service, Beaver Institute, Scottish Wildlife Trust | - Water quality changes measured directly before and after, time frame depending on canopy growth rate - Measure before and after project, control, and impact (BACI) with longer time horizon to capture change and persistence - Adaptive Silviculture for Climate Change Network; Climate Change Response Network; extant USGS gauges for temperatures | - Depends on metrics, including the frequency of climate events from which recovery is to be measured - Will need to tie to time horizons of parameters’ rates of change - Climate-ready estuaries; National Estuarine Research Reserves, Coral Reef Watch |
| VIII and IX. Analysis and results: Does data support efficacy, and at what scales/timeframes? | Measurements provide evidence that objectives of hydrologic connectivity, habitat benefits are being maintained/restored across the assessed landscape | Measurements confirm modulation of average and/or peaks of stream temperatures, and temperatures remain controlled during periods of drought | Measured indicators support that shoreline integrity is maintained or increasing as climate changes. Measured co-benefits and trade-offs (e.g., mangroves reduce wave strength but can exacerbate flooding) |
| X. Dissemination and communication   - Audience(s) - Programmatic/jurisdictional requirements - Reporting interval and methods | - Fish & wildlife agencies, ranchland managers, water regulators - Applicable water regulation protocols and regulatory compliance - Reporting intervals dependent on partner needs, target audience, and necessary programmatic and jurisdictional requirements; peer-reviewed publications, in-house reporting | - Funders; state, federal level partners, agricultural extension services - Compliance with regulatory requirements: collaboration with government agencies responsible for water quality regulations and enforcement, such as fish & wildlife agencies - Annual/final reporting as necessary for programmatic requirements; stakeholder feedback sessions, peer-reviewed publications | - Land managers, federal agencies, conservation NGOs - Establish data-sharing agreements; leverage collaborations with agencies responsible for coastal management, regulatory oversight - Reporting interval depends on objectives, programmatic and jurisdictional requirements; use data-sharing platforms, peer-reviewed publications, annual reviews, stakeholder meetings |
| XI. Conclusions: What conclusions or recommendations can be drawn from results of the proposed assessment? | - Demonstrate statistically measurable improvement of hydrologic benefits (e.g., increased water quality and supply, floodwater storage, hydrologic connectivity) - Recommend how, where to implement beaver or beaver-analog actions to support climate-smart hydrology | - Determine if canopy cover persists in modulating stream temps - How extent of temp regulation maps to ecologically significant species thresholds - Recommend how to implement forest canopy enhancements for climate-change-related water quality goals | - Demonstrate that NbS measurably increases coastal resilience to climate change - Establish return on investment for a given nature-based solutions - Recommend guidance on engineering designs and modifications as well as conditions that can enhance/impede success for a given NbS |

# 
